# Supplementary material for: Syphilis seroprevalence and risk factors among first-time blood donors in Brazil: A comprehensive repeated cross-sectional analysis spanning a decade
Source: PLoS One. 2025 Feb 21;20(2):e0315933. doi: 10.1371/journal.pone.0315933 (PMC11844829; doi:10.1371/journal.pone.0315933)
Supplement: Attachment — Submitted filename: August 29.pdf [file pone.0315933.s002.pdf]

August 29, 2024

Dear,

Syphilis is a disease that continues to haunt populations, especially in developing and underdeveloped countries. As well noted by the author of the article, advancing in the control of the disease is a current challenge and a permanent obligation of public health. As the article demonstrated, the distribution of the disease in Brazil is not uniform and is influenced by socioeconomic and geographic inequalities that result in disparities in the provision of health services. Although syphilis is not a disease confined to disadvantaged groups or classes, it has been associated with low socioeconomic status. Among these factors, low education and income levels may be significant markers, indicating difficulties in accessing healthcare services. Social inequities can result in vulnerable populations not being adequately reached and monitored by health services. Additionally, high rates are observed in non-white populations, along with a temporal trend of increasing cases among young people, especially adolescents. Studies like the one presented are important as they allow for more effective intervention measures based on the knowledge of sociodemographic, behavioral, and healthcare factors related to syphilis, so that health policies can be better directed toward the care of sexually transmitted infections.

Some points in the text are recommended for improvement.

a) It would be highly beneficial to include a detailed description of the methodology used for syphilis testing with the ARCHITECT Syphilis TP assay. As this is a central point in the analysis of seroprevalence data, including this information could add even more value to the study by providing greater transparency and allowing other researchers to reproduce and compare their results more accurately.
b) In the methodology section, it is noted that the syphilis seroprevalence among blood donors significantly decreased during the periods of 2013-2016 and 2017-2020 compared to 2007-2009, as shown in Table 3. I recommend discussing these data more thoroughly in the text, as it highlights an important trend of declining prevalence over time.
c) In the results and discussion sections, it is mentioned that syphilis seroprevalence varies significantly among the different donation centers, as shown in Table 3. Specifically, PE (Hemope) and RJ (Hemorio) present significantly higher syphilis prevalence compared to MG (Hemominas), while SP (Fundação Pró-Sangue) shows a significantly lower prevalence. I believe it is crucial to discuss these regional

variations, as they may reflect differences in infection prevalence or be influenced by sociodemographic factors specific to the blood donors in each location. A deeper analysis of these differences could provide valuable insights into the local conditions affecting syphilis seroprevalence and contribute to a more comprehensive understanding of the epidemiological landscape in Brazil.

d) The author should discuss the role of blood banks in identifying syphilis cases from a public health perspective concerning STIs, addressing how these institutions contribute to prevention, guidance, and health education, as well as their role in controlling and reducing the transmission of these infections.

I am available for any additional clarification or necessary revisions. Thank you for the opportunity to contribute to the review of this manuscript.
