# Supplementary material for: Syphilis seroprevalence and risk factors among first-time blood donors in Brazil: A comprehensive repeated cross-sectional analysis spanning a decade
Source: PLoS One. 2025 Feb 21;20(2):e0315933. doi: 10.1371/journal.pone.0315933 (PMC11844829; doi:10.1371/journal.pone.0315933)
Supplement: Attachment — Submitted filename: Response to Reviewers.docx [file pone.0315933.s001.docx]

**Response to Reviewers**

**Article Title: “Syphilis Seroprevalence and Risk Factors Among First-Time Blood Donors in Brazil:  A Comprehensive Repeated Cross-Sectional Analysis spanning a decade”**

Dear Reviewers and Editor,

Thank you for your attention to the details of our submission to PLOS ONE (PONE-D-24-14299). Regarding the discrepancy in the ethics documentation, the individual Dr. Anna Bárbara de Freitas Carneiro Proietti, named as an investigator on the ethics documents, played a foundational role in the initial stages of the research project, which began in 2006.

As the project evolved, subsequent studies, including the present submission, were developed to perform secondary analyses of the data. These studies have been carried out by different researchers, including myself, who were not part of the original project team but have utilized subsets of the data for further research. Thus, while Dr. Proietti is not an author on this current manuscript, her contributions are crucial and acknowledged in the initial setup and data collection phases of the broader project. Additionally, Ester Sabino, a senior researcher and co-author of the present study, was among the initial authors who obtained ethical approval for this study. Our use of the data for secondary analysis has been covered under the ethics approval originally granted for the project, which allows for such subsequent utilization by different investigators

In response to your request for information regarding data anonymization and informed consent, we provide the following details:

1. **Data Anonymization**:
   - All data used in our study were fully anonymized before access. The database of participating blood centers allows data to be collected anonymously, effectively avoiding any association that could identify donors and the serological results found.
   - The collected samples were de-identified after capturing the main demographic data at each collaborating blood center, ensuring it is impossible to link them with individual donors.
2. **IRB or Ethics Committee Waiver**:
   - The study protocols have been reviewed and approved by the National Research Ethics Committee (CONEP) and the respective local Research Ethics Committees (CEPs). Both protocols are exempt from the free and informed consent form (TCLE) according to Brazilian regulations.
3. **Informed Written Consent**:
   - Since the protocols are exempt from the requirement for informed consent, no patients provided written consent specifically for this study. The ethical approvals granted by CONEP included waivers for the informed consent requirement based on the retrospective nature and the anonymized handling of data.

These measures ensure the confidentiality and ethical handling of all data used in our research.

Thank you for considering our manuscript.

Andre L Cortez, MD, corresponding author

June 26th, 2024
