# Supplementary material for: Syphilis seroprevalence and risk factors among first-time blood donors in Brazil: A comprehensive repeated cross-sectional analysis spanning a decade
Source: PLoS One. 2025 Feb 21;20(2):e0315933. doi: 10.1371/journal.pone.0315933 (PMC11844829; doi:10.1371/journal.pone.0315933)
Supplement: Attachment — Submitted filename: Answering to Reviewers (PLos One).docx [file pone.0315933.s003.docx]

**ANS****WERING TO THE REVIEWERS**

October 30, 2024

Attention: Section Editor of the *PLos One*

Subject: Manuscript’s revision PONE-D-24-14299

Dear Albert Schriefer:

Thank you for your kind reply. We cautiously reviewed all commentaries and suggestions, and we are sure they contributed to improving the quality of our work. Please find below our answers to the reviewers' comments. I am attaching a highlighted version of the revision with all changes marked and a final, clean version of the manuscript.

We look forward to hearing from you soon.

Sincerely,

**Dr. Andre L. Cortez**

**ANSWERS TO REVIEWERS**

**We are very thankful for the suggestions and comments that improved the manuscript.**

**Reviewer 1**

*Introduction.*

- *Add the reference [4] for “The incidence of acquired syphilis among adolescents (aged 13 to 19) has increased 2.2-fold between 2015 and 2021, according to recent data released in 2022” and remove it from the next sentence.*

**The reference was reallocated.**

- *In the sentence “Routine screening in Brazil includes serological tests for HIV, hepatitis B (HBV, both core antibodies [HBcAb] and surface antigen [HBsAg]), hepatitis C (HCV), human T-lymphotropic viruses (HTLV)-1 and -2, Trypanosoma cruzi (Chagas disease), and syphilis”, add “T. pallidum” before “syphilis” and put the latter within brackets, as you did for T. cruzi, to maintain consistency of listing the pathogens first and the diseases they cause second.*

**The sentence was adjusted. Thank you for this observation.**

- *Add “being” after “than” in “In the context of syphilis, the detection of antibodies through these treponemal methods signifies a history of exposure to the pathogen rather than indicative of an ongoing active syphilis infection [6]” or rephrase in another way.*

**The sentence was reformulated. Please check if now is more precise.**

*Statistical methods*

- *You say “To determine syphilis seroprevalence, the total number of syphilis-positive blood donors without any previous donation record was considered the numerator, with the total number of first-time blood donors with valid treponemal results being the denominator.” Did you consider calculating syphilis incidence for first-time donors by extrapolating from the repeat donor incidence – a method already used in transfusion risk research? The trouble with syphilis seroprevalence is that it cannot inform how recently the infection occurred. In contrast, incidence estimation with a person-year denominator goes directly at the heart of your research question on secular trends. Although you can infer syphilis incidence from sequential prevalence estimates, it is an indirect and less precise method as it lacks the infection recency parameter. Older donors had longer exposure to T. Pallidum, but you cannot tell at what age they acquired the infection from the prevalence data. For younger donors, this uncertainty is less acute, but it may still be years or even more than a decade apart between the infection and blood donation. This is something to be addressed in the discussion.*

In response, we have revised the discussion section to explicitly address this issue.

We recognize that using a person-year denominator for incidence estimation would offer better insights into recent syphilis trends. However, modeling incidence from repeat donors poses challenges, including donor selection bias (as repeat donors often represent a healthier population) and difficulties in tracking infection timing without comprehensive follow-up data. Despite these limitations, our group is planning to conduct this analysis in a separate study to further explore syphilis incidence trends

- *“We excluded serology results as covariates to avoid the risk of multicollinearity” sounds odd. Why would rare co-infections, typically with HBV and/or HIV, induce collinearity?*

Our rationale for excluding serology results as covariates was based on the potential overlap in risk factors between syphilis and co-infections, such as HBV, HCV, and HIV, which could introduce redundancy and multicollinearity in the model. As shown in Table 2, seroprevalence rates of these infections, though lower than syphilis, still share many common risk factors, such as sexual behavior, socioeconomic status, and healthcare access. For instance, the syphilis seroprevalence among HIV-positive individuals was significantly higher, ranging from 9.21% to 16.13% across centers.

- *You say “Subsequently, model comparisons were conducted using an Analysis of Variance (ANOVA) to test the significance of the added interaction term, with a p-value threshold of 0.05”, but it seems at odds with Poisson regression mentioned earlier. Was it a post-hoc regression analysis or was the term included in the regression model? Can you write down the equation? Which software did you use?*

Based on the reviewer’s comments, we revised our methodology to better evaluate the interaction effect. To assess the significance of the interaction term, we compared nested models—one with the interaction term and one without it—using a likelihood ratio tests (LRT), which is preferred over ANOVA for generalized linear models like Poisson regression, providing a more accurate assessment of improvements in model fit.

Here is the code snippet for the equation used in R:

\# Perform the likelihood ratio test (LRT) for model comparison

model_comparison <- anova(model_without_interaction, model_with_interaction, test = "LRT")

***Methods and Results Sections were corrected to reflect the corrected approach adopted.***

*Results*

- *Define the algorithm for “valid serological results for syphilis”. The donors with either treponemic or non-treponemic test results were discarded from the statistical analysis; if so, how different were their sociodemographic characteristics and serologic testing results from those included in the study?*

Treponemal testing began at different times across locations, impacting the availability of consistent serological data. In Pernambuco (PE), treponemal testing was implemented in 2010, allowing us to include data from 2010 onward. In Rio de Janeiro (RJ), treponemal testing started in the end of 2012, so data from this location were included beginning in 2013.

Following this location-based selection, we included only donors with definitive positive or negative syphilis test results, excluding records with undetermined or unknown results to enhance data clarity. This last exclusion step affected a small portion of the dataset, comprising only 4% of the initial sample. Given the expected minimal impact on the overall sample, we did not analyze or describe the sociodemographic characteristics of these excluded records separately.

- *In Table 2, the phrase “Estimates of Syphilis Seroprevalence Rates” should drop the word “Rates” because it implies a person-time denominator, which was not used here.*

**The sentence was adjusted. Thank you for this observation**

- *In Table 3, “ref.” should be substituted by “1.00”. Although the value is implied in the seroprevalence ratio description, it is better to state it explicitly than to keep some readers guessing.*

**The table was adjusted. Thank you for this observation**

- *“Predicted Syphilis Seroprevalences” in Figure 4 are Poisson model-based marginal means or proportions?*

To illustrate the interaction between visit period and age group, we generated marginal predictions of syphilis seroprevalence for each combination of age group and visit period using the fitted Poisson regression model. We employed the emmeans R package to compute these marginal means, which represent predicted syphilis seroprevalences averaged across levels of other predictors in the model.

*marginal_means_interaction <- emmeans(model2, ~ age_group * visitpr, type = "response")*

***Methods section was updated to clarify this question.***

- *It is unclear to me what was meant by “According to the model, despite Figure 4, there is an increase in prevalence among persons aged 18–34”. Why “despite Figure 4”? Also, you say “The decrease in overall prevalence in recent years is to be attributed to the decline in older strata”, and I suppose this means that the older age groups reduced their prevalence over time as opposed to the younger blood donors. Please clarify and rephrase.*

Thank you for pointing this out, and we apologize for the lack of clarity. Our intention was to convey that Figure 4 visually reflects an overall decreasing trend in syphilis seroprevalence, particularly pronounced among older age groups (such as those aged 55–65). However, the model also indicates a slight upward trend in seroprevalence over time among younger age groups (18–34).

**We have rephrased this section for clarity.**

*Discussion*

- *In the reference [8], the overall syphilis seroprevalence was 0.14%, not 0.13% as reported in the present study. More importantly, the prevalence was 0.19% among the first-time donors, so this is the value directly comparable with the present study prevalence. This is important in light of your statement “A possible explanation for the higher prevalence in our analysis is that we only considered first-time donors, thus reducing the selection bias and dilution effect of including repeat donors, as only seronegative blood donors would be invited to donate again.” Other southern Brazilian states (e.g. Paraná) also reported lower syphilis seroprevalence among first-time blood donors [8], so systematic regional variations may be a more plausible explanation.*

**Thanks for this correction. The mentioned citation was corrected.**

- *Also, the two-step blood screening that starts with a treponemal test, followed by a non-treponemal test, hugely reduced false positive donor deferral in Brazil, thus reducing the “dilution effect of including repeat donors” (Baião A. et al. in Transfusion Medicine 2014; 24(1):64-66, doi: 10.1111/tme.12095). Resuming, a more thorough discussion of alternative explanations is due.*

**...**

- *Age-by-sex secular trends, adjusted for other covariates in multivariable Poisson regression, should be added to better understand how and for whom these trends are changing over time. This is equivalent to adding sex to the interaction term or just a post-hoc regression analysis of marginal means for the age-by-sex groups.*

Thank you for this suggestion. In our primary analysis, we evaluated the interaction between age group and visit period within a multivariable Poisson regression model to understand syphilis seroprevalence trends by age over time. Following peer review feedback, we conducted post-hoc analyses incorporating additional interaction terms to examine syphilis seroprevalence trends by visit period in relation to sex, self-reported ethnicity, and educational level, each tested separately. For each model, we generated marginal means for syphilis seroprevalence by subgroup, adjusted for other covariates. Model fit improvements were assessed using Likelihood Ratio Tests (LRT) and AIC values, which confirmed statistically significant effects for each interaction term. Fig 4 presents these predicted marginal means, illustrating how seroprevalence trends vary by age, sex, ethnicity, and educational level over time

- *The inclusion of only first-time blood donors in statistical analysis was considered a “notable strength” by the authors. However, it has many drawbacks in comparison with incidence estimation, already mentioned in my comments on statistical methods. Although the incidence estimation methods for first-time donors have their caveats, they rest on solid statistical principles, like so-called raking. I could not find sufficient arguments for favoring serial prevalence over incidence in the present study, nor did I see why first-time donors would be “a more reliable sentinel surveillance group for what might happen epidemiologically in the general population [21].” Behavioral factors are hugely important in motivating people to donate blood and hold the key to the understanding of donor candidate (auto)selection. Resuming, I suggest a better elaboration of the arguments stated for the cited recommendation.*

**We have removed the statement suggesting that the selection of 'first-time blood donors' is a study strength.**

We acknowledge that incidence measures may offer more direct insights into transmission dynamics. Incorporating additional interaction effects, as suggested during the peer review process, enabled us to explore syphilis seroprevalence trends across various demographic subgroups.

**Dear Reviewer, thank you for your kind feedback. We appreciate it. Your comments and suggestions will undoubtedly improve the quality of our paper.**

**Reviewer 2**

- *The study was conducted with rigor, utilizing a repeated cross-sectional and multicenter design, which allowed for the collection of data from a large sample of blood donors over a significant period (2007-2020). The inclusion of over 1.4 million first-time donors strengthens the robustness of the data. The use of multivariate Poisson models, including interaction terms, demonstrates an advanced and appropriate statistical approach to analyze syphilis seroprevalence and identify associated risk factors. The comparison of models using ANOVA reinforces the validity of the analyses. The conclusions are drawn from the presented data, such as the increase in syphilis prevalence among younger donors, particularly those born after 1990, indicating a concerning trend that requires continuous surveillance. Overall, the manuscript meets the criteria for technically sound scientific research, with data that adequately support the conclusions.*
- *Yes, the statistical analysis was performed appropriately and rigorously. The study utilized multivariate Poisson models, which are suitable for analyzing syphilis seroprevalence and identifying associated risk factors. The inclusion of interaction terms, such as between age group and donation year, adds depth to the analysis and allows for a more nuanced interpretation of trends over time. Furthermore, the model comparisons using ANOVA to evaluate the significance of these interaction effects demonstrate a rigorous approach. The improvement in the adjusted R² from 0.10 to 0.11 and the associated p-value of less than 0.001 reflect the robustness of the statistical analysis.*

**Dear Reviewer, thank you for your kind comments. We appreciate your positive feedback.**
